# Function of miR-152 as tumor suppressor in oral squamous cell carcinoma cells by targeting c-MET

**DOI:** 10.3892/or.2022.8254

**Published:** 2022-01-04

**Authors:** Minghe Li, Zhihong Li, Xue Wang, Yumei Wang, Cong Zhao, Lei Wang

Oncol Rep 39: 1173-1180, 2018; DOI: 10.3892/or.2017.6157

Following the publication of this paper, it was drawn to the Editors’ attention by a concerned reader that certain of the representative tumor images shown in Fig. 6B were strikingly similar to images appearing in different form in another article by different authors. Subsequently to the comments raised by the reader, the authors themselves contacted the Editorial Office to request a retraction of the above paper on account of not being to reproduce the results shown in the *in vivo* experiments.

Owing to the fact that the contentious data in the above article had already been published elsewhere prior to its submission to *Oncology Reports*, and in line with the authors’ own request, the Editor has decided that this paper should be retracted from the Journal. The Editor apologizes to the readership for any inconvenience caused.

